# It Is Necessary to Purpose an Add-on to the American Classification of Endometriosis? This Disease Can Be Compared to a Malignant Proliferation While Remaining Benign in Most Cases. EndoGram® Is a New Profile Witness of Its Evolutionary Potential

**DOI:** 10.3389/fsurg.2019.00027

**Published:** 2019-06-07

**Authors:** Jean Bouquet de Joliniere, Attila Major, Jean Marc Ayoubi, Rosalie Cabry, Fathi Khomsi, Guy Lesec, René Frydman, Anis Feki

**Affiliations:** ^1^Department of Gynecology and Obstetrics, HFR, Fribourg Cantonal Hospital, Friborg, Switzerland; ^2^Department of Gynecology and Obstetrics, Foch Hospital, Suresnes, France; ^3^Department of Anatomopathology, Unilabs, Lausanne, Switzerland

**Keywords:** endometriosis, AFS classification, phenotype, genotype, breast cancer

## Abstract

Endometriosis is a curious pathology that has been the topic of many international publications. Its etiology remains mysterious but seems to have multiple causes. It is a complex disease whose lesions are very heterogeneous in where they can occur (deep endometriosis, superficial, ovarian cyst), extent, associated symptoms, evolution or aggressiveness of the disease, and response to treatments. Furthermore, it evolves in pushes, remains autonomous, and is responsible for both superficial and deep lesions that explain its two most well know challenges: pain and infertility. It has always been classified by the size of its anatomical lesions—Acosta classification (1), revised by the American fertility society (AFS) (2), and the American society of reproductive medicine (ASRM) classification with a description of the disease at different stages: minimal (score of 1 to 5), mild (3–12), moderate (16 to 40), and severe (>40) (13). If this classification provides a complete repertoire of implants (anatomic) (10), the attribution of points is arbitrary. In fact, the size of the lesions is not synonymous with the difficulty to treat them surgically. Their location, if deep, is larger than the size of ovarian endometriomas. In addition, small anatomical but evaluative lesions will have a larger impact than big fibrous and stable lesions (Figure 1). Thus, attempts to explain their inflammatory side effects have been proposed (14, 15). The French classification nodule, ovaries, adhesions, tube, and inflammation (FOATI) (10) has had the merit of taking this phenomenon into account. In our opinion, we must go much further and propose an amendment in this classification, taking into account the evolution of the lesions and their deep molecular biology because in reality, the lesions are not at the same stage.

We have begun to demonstrate (7) an embryological origin, chromosomal instability, as well as genomic and proteomic abnormalities (16–18). These problems are related to pharmacologic testing during a wild hormone therapy that does not take into account the phenotype of lesions. Indeed, it is possible using a common model, breast cancer. An endometriosis profile is necessary to know its phenotype, such as hormone receptors, proliferation of rank, Mib-1or (Ki 67%), growth factors, and oncogenic factors (17–19).

**Figure 1:**
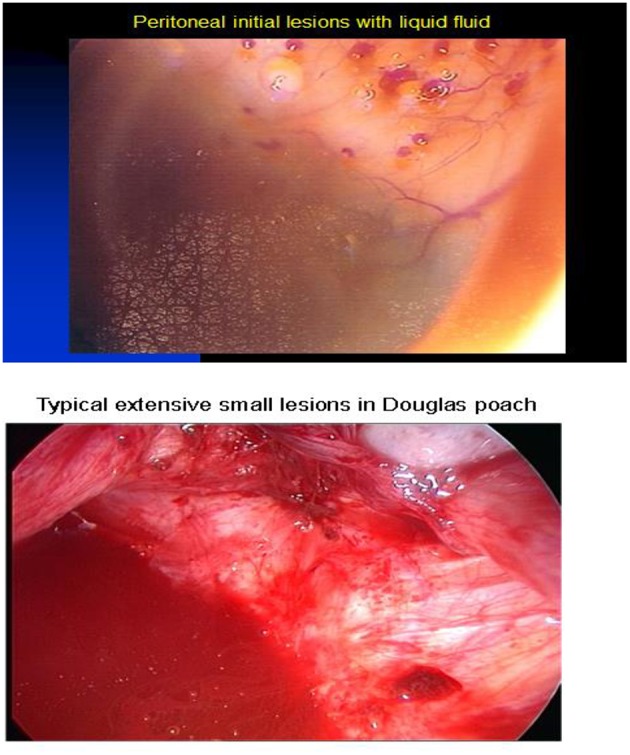
Small inflammatory lesions of endometriosis.

The peritoneal fluid is one of the factors in endometriosis diffusion in the ovaries (6, 19), deep forms under and peritoneal. In order to address the need of improving endometriosis diagnosis and management, we have developed EndoGram®, a prognostic test based on a signature of 14 biomarkers and validated in a first prospective study. For each patient, it allows us to determine: (1) the risk of recurrence of the disease after 2 years in order to identify the patients with a high risk of recurrence from the first diagnostic surgery, and to adapt their follow-up treatment to better detect the recurrence of the disease; (2) the presence or absence of the receptors targeted by the hormone treatments used at the present time—this information on hormonal sensitivity will serve as a decision-making aid for the surgeon to prescribe the most appropriate and effective therapeutic strategy; and (3) the best fertility strategy if the patient wishes to become pregnant. For the last one, depending on the age of the patient and the profile of her lesion as defined by the EndoGram® test, the surgeon will be able to optimize the patient's fertility strategy and reduce the number of failed *in vitro* Fecundation occurrences. Thus, some minimal anatomical forms are very aggressive with infertility, medication ineffectiveness, and persistence/recurrence despite surgery (19), where large ovarian cysts accessible to laparoscopic surgery do not reoffend. This makes the surgical diagnosis of the disease and its management difficult, as well as still too dependent on the experience and dexterity of the surgeon.

To meet this clearly identified need, the EndoGram® program has the aim of identifying specific markers of this heterogeneity and giving a unique and personal photograph of the stage of endometriosis for each patient, through an innovative analysis of the biopsies taken during diagnostic surgery. This information can then be used by the surgeon to adjust the therapeutic approach in particular. This article reveals all the characteristics of the disease for each patient. This article allows us to define a therapeutic attitude whose primary goal is not to let the time of the *in vitro* fertilization program (IVF) pass in young patients, and to respect the recommendations of the European Society of human reproductive medicine (ESHRE) (11) on a single, non-aggressive surgery that respects the ovaries and their follicular count. It should also be noted that this proliferative side explains that pregnancy and menopause do not cure the disease, but can only improve it. New molecules can be used according to this profile.

## Different Classifications for Endometriosis

Endometriosis is a major and disabling gynecological disorder affecting 10% of women of reproductive age, or 180 million women worldwide. It is caused by tissue similar to the endometrium that develops out of the uterus and colonizes the abdominal cavity, sometimes with involvement in the lungs. Moreover, it results in severe and chronic pain that is oftentimes unsustainable, causing infertility in more than 40% of women with endometriosis. It is a debilitating and costly disease that represents an annual expenditure of 30 billion euros in Europe (written statement on endometriosis in the European Parliament 2005) and 49.6 billion dollars in the United States (20). Despite these alarming figures, there is no definitive treatment for the disease. Most of the time, the therapeutic strategy involves combining surgery with hormone therapy. Thus, diagnostic surgery is also therapeutic with the removal of all visible lesions.

It is therefore necessary to create a classification to assess the stage of the lesions and thus, better treat them. Before ASRM or revised AFS classifications, many of them were proposed by Wicks and Larson (12), Huffman (21), Acosta et al. (1), Kirstner et al. (22), and Buttram (23). In 1979, the American Fertility Society created the AFS classification (13), followed by a revised form in 1985 (2). In 1996, the committee of the American Society for Reproductive Medicine (ASRM) added to the revised classification a description of peritoneal implants (red, white, and black) (10) (Figure 4) with different grades and defined scores: minimal (scores 1–5), mild (6–15), moderate (16–40), and severe (>40) (Figures 4, 5). However, these scores are arbitrary. The given points do not take into account inflammatory phenomena (Figure 5). We know that small, progressive lesions can be much more aggressive in the pelvis and “harmful” to fertility than giant, calm anatomical lesions. Indeed, it is easy to understand why, in the literature, there is so much discordance in treatments and results because the lesions do not have all the same characteristics (10). Adamson and Pasta proposed an index of endometriosis to predict pregnancy rate using IVF program (24). Finally, Tran and Belaisch (10) developed the FOATI classification in 1991–1992, which became FOATI ARVS (F, foci for superficial implants; O, ovarian endometrioma; A, adhesions; T, lesions of the tubal wall; I, inflammatory appearance; A, adenomyosis; RVS, involvement of the recto vaginal wall) in 2010 after revision (Figure 3). Therein, points are given as 0, 1, and 2, and this classification gives 2 points for functional consequences of lesions (Figure 6).

The need for a new profile that takes into account not just the size of the lesions, but also their evolution potential is closer to reality. Endometriosis is a dynamic disease in the sense that time is its partner. Its phenotypic potential becomes a considerable help for the administration of hormonal treatments, for the decision of PMA, for the preservation of fertility factors, for the quietness of the couple, and for a satisfying and flexible therapeutic approach near the pathogenesis.

## What is EndoGram®?

### Concept and Approach

The EndoGram® project began in 1995 on the basis of the fact that endometriosis is a complex disease whose lesions are very heterogeneous on the part of their location (deep endometriosis, superficial, ovarian cyst, etc.), extent, associated symptoms, the evolution or aggressiveness of the disease, and response to treatments. Thus, some minimal anatomical forms are very aggressive with infertility, medication ineffectiveness, and persistence/recurrence despite surgery (25–27), where large ovarian cysts accessible to laparoscopic surgery do not reoffend. This makes the surgical diagnosis of the disease and its management difficult, as well as still too dependent on the experience and dexterity of the surgeon. To meet this clearly identified need, the EndoGram® program aims to identify specific markers of this heterogeneity and give a unique and personal image of the stage of endometriosis for each patient, through an innovative analysis of the biopsies taken during diagnostic surgery. This information can then be used by the surgeon to adjust the therapeutic approach and, in particular, to determine for each patient the risk of recurrence and its hormonal sensitivity.

The rate of recurrence, however, remains very high (about 50% in 2 years) and for some patients, unfortunately more than a dozen operations are performed. These surgeries are often extreme and radical with organ ablations (bladder, terminal colon, hysterectomy, and oophorectomy), risks of major complications, and increased morbidity. The chances of conceiving after the second surgery are half as high as after the first surgery due to a decrease in the ovarian reserve (28). Once the surgical diagnosis and treatment are made, different hormonal treatments can be proposed. However, these treatments are not specific, are ineffective, and often associated with many serious side effects (loss of bone density, virilization, acne, weight gain, depression). Therefore, it is imperative to not be aggressive, to comply with ESHRE recommendations (chapter 5), and to preserve the fertility of these patients by using mild solutions, by reserving laparoscopy for the diagnosis, sampling, and profile of the disease.

### EndoGram® Proposes a Unique Analysis of Endometriotic Tissues, an Expertise for a New Therapeutic Strategy (Figure 2)

The examination of the lesions arose from a comparison between endometriosis and breast cancer. It is possible to identify the lesions before a therapeutic approach through grade, MIB-1 (Ki 67%), phenotype with progesterone receptor assays, estrogen, androgen, and oncogenic and proliferation factors. The peritoneal fluid is rich in factors (Her2/neu, Myc, etc.). Moreover, it behaves like the peritoneal vector of dissemination of the disease, promotes angiogenesis and implantation of lesions, and recovers the protein information after apoptosis to allow young lesions to express themselves.

**Figure 2:**
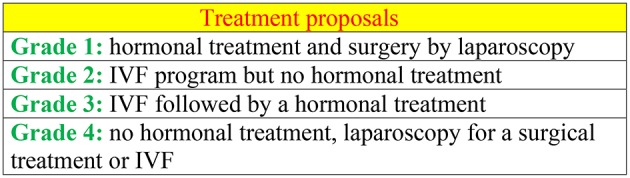
Treatment according to the stage of Endogram.

EndoGram® defines an endometriogram, a real ID card of the disease, evolution of the disease, and aggressiveness label. In addition, the EndoGram® report includes cell line proliferation test (16), phenotype analysis using immunohistochemistry for each specimen, genotype analysis (16–18), histology glass slide pictures, and aggressiveness and progression scores of the disease. Endogram® also provides surgeons with detailed reports that significantly add to the clinical information currently used to determine which therapeutic options are considered. Furthermore, it will be complementary to the AFS classification.

That research relied on a first series of 35 biomarkers identified by the scientific founders of the company, who have spent more than 20 years working on this disease, and by the academic team's partners. The precise nature of these biomarkers is kept confidential. The markers of interest belong to the families below.

- Angiogenesis. The vascular endothelial growth factor (VEGF) is a key mediator of angiogenesis. It is suggested that it contributes to the development of endometriosis by encouraging the neovascularization of endometrial cells that invade the peritoneum.
- Inflammation/cytokines- One of the main characteristics of endometriosis is its inflammatory nature (Figure 6). It has been shown that cytokines released by immune cells play an important role in the pathogenesis of endometriosis (29).
- Immunology-Endometriosis is associated with changes in cellular and humoral immunity. Natural killer cells exhibit inhibited activity with a defect in the elimination of refluxed menstrual debris. Macrophages are hyper activated and secrete growth factors and pro-inflammatory mediators that stimulate cell proliferation.
- Hormones-Estradiol is found in high concentrations in endometriotic lesions (30). Estrogen is a powerful stimulus of angiogenesis by a direct increase in VEGF expression (31, 32). The 17β-HSD enzymatic defect responsible for the conversion of estradiol to Estrone leads to an accumulation of estradiol in endometriotic lesions (33).
- Proliferation/invasiveness-Endometriotic cells are less sensitive to apoptosis, which could promote their spread and implantation at ectopic sites (34). Overexpression of estrogen receptors (ER), receptor resistance to progesterone, and deregulation of NF-kappa activation leads to increased proliferation of endometrial cells, inflammation, and angiogenesis (35).
- Stem cells-Stem cell markers (CD9, CD34, c-Kit, and Oct-4) are present in endometriotic lesions that, in particular, should be determined for each patient the risk of recurrence and hormonal sensitivity.

Currently, in view of the therapeutic excrements practiced in endometriosis, this biological profile is a true mapping of the disease and a true personalized medicine, making possible the time of IVF to give the patient every chance for its fertility. Of note, the surgery must comply with ESHRE recommendations, and the hormonal treatment must follow the results about receptors.

## Hypothesis, a Warburg Effect?

**Figure 3:**
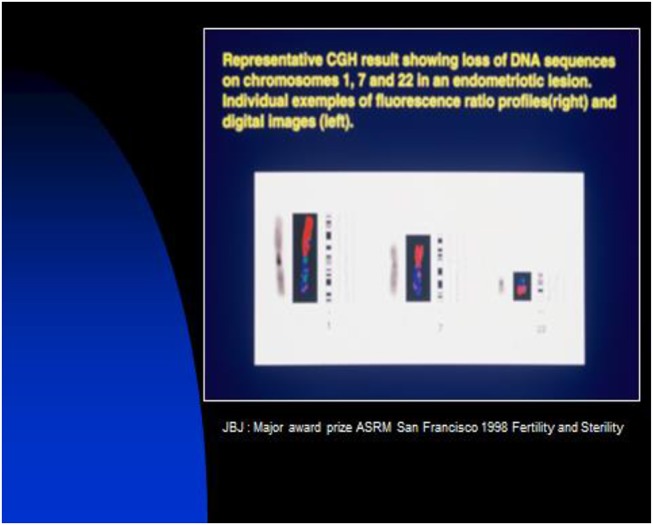
Genomic abnormalities in most endometriosis implants.

The origin of the disease remains obscure. However, a possible embryological origin has been demonstrated by a preliminary study (7). The need to define proliferation markers is related to previous studies of the causes of proliferation (4, 7, and 9). The lesions have genetic abnormalities (36) that are found statistically in almost all lesions: damage found by chromosomal instability of the nucleus DNA and on the p and q arms of chromosomes 1p, 7p, and 22q (6) (Figure 3). Furthermore, the chromosomal instability is an alteration of the chromosome constitution occurring in various pathological conditions, such as the fundamental property of neoplastic cells, precancerous lesions, chronic inflammatory conditions, infectious diseases, and diseases induced by viruses (herpes, Human Papilloma Virus, Epstein Barr Virus).

The genomic instability appears in two different types: (1) chromosomal alterations in non-neoplastic precursor lesions and mutation of the P53 gene, and (2) errors in DNA replication detected by microsatellite instability (deficiency in DNA mismatch repair mechanism). For endometriosis, we have observed such instability with chromosome number changes (4), chromosomal deletions (6), translocations, and point mutations in particular genes, nuclear DNA content, telomerase function, errors in DNA replication (18, 19), presence of endo mitosis, premature centromere disjunctions (PCD), and micronuclei.

In a previous publication (6), the authors showed a loss of heterozygosity (LOH). These studies have been conducted using DNA from histologically homogeneous endometriotic tissues. Forty lesions were studied, wherein the authors found that inactivation of tumor suppressor gene(s) may play a role in the development of endometriosis. Fluorescence *in situ* hybridization (Fish) (37) analysis has revealed more clonal aberrations than conventional cytogenetic analysis in a number of altered tissues (38). However, the comparative genomic hybridization (CGH) is the best test as a molecular cytogenetic method able to discover and map genomic regions for chromosomal gains and/or losses in a single experiment (39). Regions showing an increased copy number (gain or amplification) may harbor dominant oncogenes, whereas regions with a decreased copy number (loss) may contain tumor suppressor genes (6). Therefore, it is the loss of either essential genes or even entire chromosomes that explains the high invasive potential of the endometriotic cells. Genomic alterations (rearrangements) initiated by telomere dysfunction, for instance, can be a primary event that facilitates endometriosis initiation and spread (18). In another publication (40), the authors showed that an aerobic glycolysis marker expression is increased in endometriosis lesions compared to eutopic endometrium and in the peritoneum of women with endometriosis compared to women without endometriosis.

## Other Hypothesis

In the beginning, there are ectopic endometrial cells derived from cells having embryologic origin (7) that missed migration to the urogenital sinus. These cells will therefore stay in an ectopic location. The other well-known cause is the retrograde flow of cells in the peritoneal cavity during menstruation. In 80–90% of women, retrograde menstruation is observed (41), but compared to these numbers, only 10% of the female population present endometrioses. Endometrioses may be induced by mesenchymal cells, stem cells, or endometrial tissue (42, 43). All these cells will initiate a reaction of the immune system. This reaction will be different depending on its origin and be influenced by the genetics of the cells.

**Figure 4:**
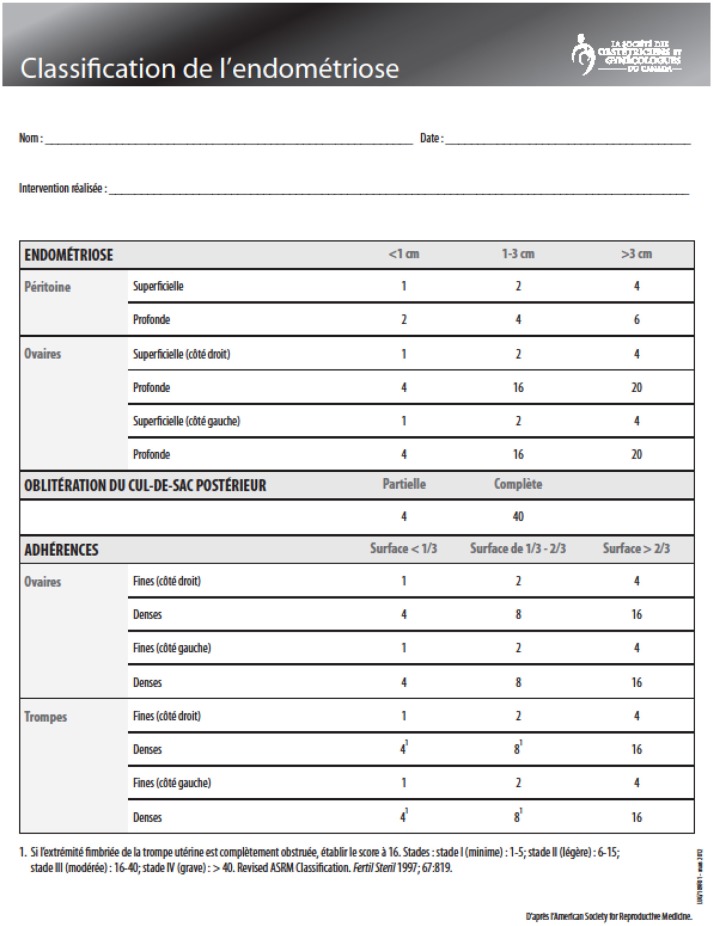
ASRM classification.

**Figure 5:**
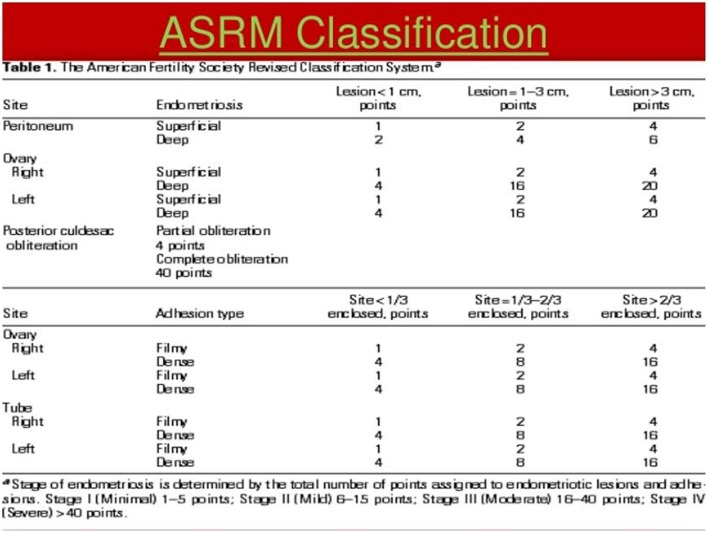
With ASRM Classification agreement. In this anatomical classification, points are given according to the size and location of the lesions to obtain a gravity score.

**Figure 6:**
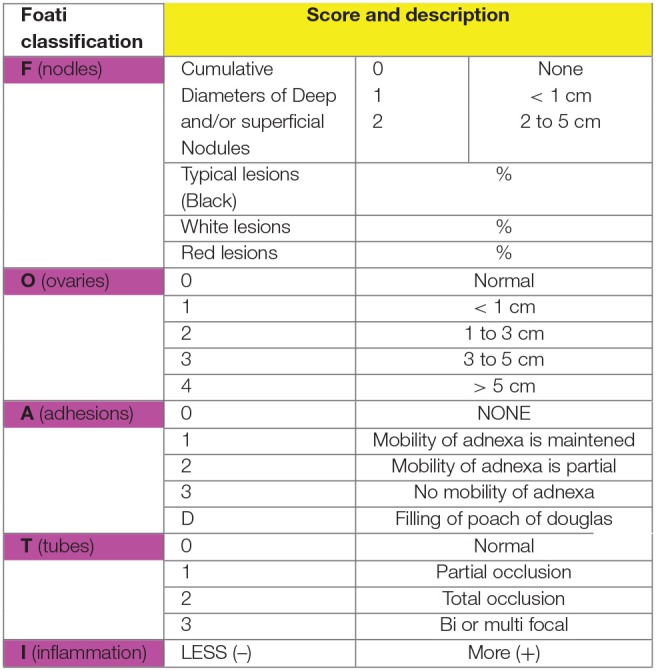
Foati classification.

Once puberty starts with the release of sexual hormones, ectopic endometrial cells are stimulated and even if they are in a vascularly unfavorable place, they start to propagate. This event initiates several processes in a cascading manner. First, production of reactive oxygen species (ROS) is stimulated by an increased metabolic turnover of cells and activation of factors for angiogenesis, attracting stem cells for neovascularization through cell signaling by ROS. Second, oxidative stress is provoked by several factors including stimulation by sexual hormones in concert with propagation of cells (increased energy production in mitochondria), immune reaction of the peritoneum (an immunologic reactive organ), and degradation of hemoglobin and toxic effect of iron by the Fenton reaction. ROS production itself serves as a stimulus by cell signaling for more propagation and immune reactions with a positive feedback mechanism potentiating it. Third, an important misbalance between ROS and the anti-oxidative defense mechanism of the cell becomes toxic and induces chromosome instability. If the shock is significant, DNA damage can occur followed by necrobiosis. Finally, all these processes can change the cell metabolism and induce aerobic glycolysis (43). This switch, termed Warburg effect, is to satisfy the needs for structural molecules like lipids, proteins, and nucleic acids, and at the same time, to diminish oxidative phosphorylation to protect the cell from the damaging effect of ROS by decreasing the production of ROS.

In certain circumstances, inflammation, acidosis, and continuous DNA damage by ROS can even drive endometrioses to malignant transformation (42), depending on four factors: origin of cells; reaction of the immune system; location of ectopic endometrial cells; and ingestion of hormones and toxic molecules. All these factors interact with each other and drive a new balance, which will be different depending on the staging of endometriosis and the endogram. Endometriosis causes important inflammation by the interaction with the environment, thereby increasing ROS production (44–46). In turn, ROS induces DNA damage, while endometriosis produces cytokines. Unfortunately, there is no efficient and causal treatment for endometriosis. This makes the surgical diagnosis of the disease and its management difficult, in addition to problem that the effectiveness of a surgical intervention is still too dependent on the experience and dexterity of the surgeon. To meet this clearly identified need, Endodiag has developed the EndoGram® program with the aim of identifying specific markers of this heterogeneity and giving a unique personal photograph of the stage of endometriosis for each patient, through an innovative analysis of the biopsies taken during diagnostic surgery.

## Discussion

The discussion is how to use this data in practice. The objective will be a prospective, multi-centric international study to confirm, on a large scale, the selection of a combination of biological markers, in order to first establish a diagnosis of endometriosis from an endometrial biopsy. Moreover, codifying the disease in stages (19) makes it possible both to avoid mistakes in medical treatment (such as giving progestogen alone to treat endometriosis with negative receptors) and to perform aggressive surgery when there is an indication of IVF in a woman whose age changes in her forties and whose anti Mullerian Hormone (AMH) is no longer satisfactory.

## ESHRE Recommendations for Care

EndoGram® is perfectly in alignment with the recommendations of the ESHRE, and allows us decide the time of the IVF, whether to operate surgically in order to respect the rules of a gentle management of the lesions described by ESHRE (this will, by limiting the adhesions, respect the pelvic anatomy as much as possible and thus limit the pain), and to not give hormones or progestin alone in the hormono-independent forms.

### Management of Ovarian Endometriomas

#### For the Fertility

The main issue is the management of a woman with ovarian endometrioma who is considering using IVF. The major concern is whether the resection of endometrioma leads to the loss of small follicles adjacent to the cyst wall, with the consequence being a reduction in ovarian reserve and ovulation frequency in the ovary undergoing surgery. Although there is no consensus on the best approach, most fertility specialists would agree not to excise these lesions before starting IVF, because there is no clearly demonstratable advantage, and ovarian surgery can decrease ovarian function. It is preferable to resect endometriomas before IVF only for specific indications such as pain or suspicion of ovarian cancer, or if the size and/or location of the endometrioma would limit the oocyte puncture in a patient. The Study group of endometriosis (GEE) (15) recommends that women with endometrioma be advised about the risk of decreased ovarian function and reserve after surgery, as well as possible loss of ovaries. Moreover, the decision to proceed with surgery should be carefully considered if the woman has had a history of ovarian surgery.

In infertile women receiving endometrioma surgery, clinicians should perform excision of the endometrioma capsule, instead of drainage or electrocoagulation of the endometrioma wall, which can increase the rates of spontaneous pregnancy. Moreover, for those with AFS/ASRM stage I/II endometriosis, clinicians may perform an intra uterine insemination (IUI) with controlled ovarian stimulation instead of expectancy because this treatment increases the live birth rate, and instead of UTI without ovarian stimulation as treatment increases the rate of pregnancy. Clinicians may also consider intra uterine insemination (IUI) with controlled ovarian stimulation within 6 months after surgical treatment, since pregnancy rates are similar to those obtained in the case of unexplained infertility. For treatment after surgery, women can be prescribed with ART if cumulative rates of recurrence of endometriosis do not increase following controlled ovarian stimulation associated with IVF/ICSI. Clinicians may also use antibiotic prophylaxis at the time of oocyte retrieval, although the risk of ovarian abscess following follicular aspiration is low. In this case, they can prescribe GnRH agonists for a period of 3–6 months before ART treatment to improve the rate of clinical pregnancies in infertile women with endometriosis. What concerns the results in terms of pregnancy? The effectiveness of surgical excision of deep nodules prior to ART treatment is not well established. In infertile women with stage III/IV (AFS/ASRM) endometriosis, clinicians may consider laparoscopic surgery instead of an expectant attitude to increase rates of spontaneous pregnancy.

#### For Pain

During surgery in women with ovarian endometrioma, clinicians should perform cystectomy instead of drainage or electrocoagulation because it reduces the pain associated with endometriosis. Clinicians may also perform cystectomy rather than CO_2_ laser vaporization due to a subsequent low recurrence rate. In women with endometrioma ≥3 cm, ovarian cystectomy should be carried out instead of drainage or electrocoagulation, to prevent secondary endometriosis associated with dysmenorrhea, dyspareunia, and non-menstrual pelvic pain. After a cystectomy for ovarian endometrioma in women who have no desire for conception, clinicians are advised to prescribe hormonal contraceptives for the secondary prevention of endometrioma. Post-operation, women can be prescribed with a levonorgestrel IUD or combined hormonal contraceptive for at least 18–24 months. This is one of the options for secondary prevention of dysmenorrhea associated with endometriosis, but this is not an option for pelvic pain or non-menstrual dyspareunia.

**Figure 7:**
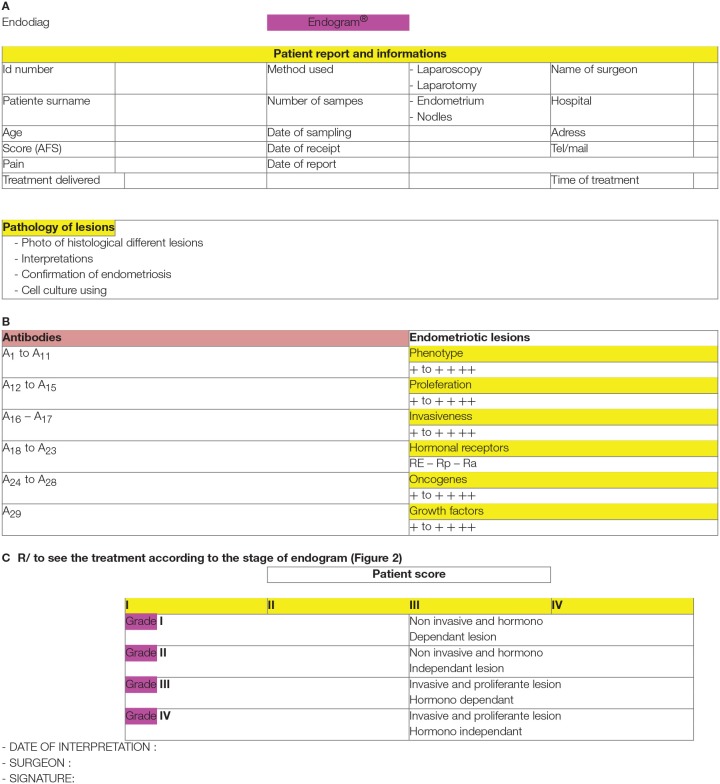
**(A–C)** Grade classification 1 to 4 for endometriosis depending of hormonal receptors and proliferation factors.

#### Are Hormonotherapy Effective for Infertility Associated With Endometriosis?

In infertile women with endometriosis, clinicians should not prescribe hormone therapy for the suppression of ovarian function in order to improve fertility. For those with AFS/ASRM stage I/II endometriosis, clinicians must perform excision or ablation of endometriosis lesions, including adhesiolysis, rather than only diagnostic laparoscopy, to increase the pregnancy rate. Moreover, they may consider a CO_2_ laser vaporization of endometriosis instead of electrocoagulation, since laser vaporization is associated with higher cumulative rates of spontaneous pregnancies (47).

#### The Risks of Malignancy

It is still the specialist of anatomo-pathology, with immunohistochemistry and molecular biology, who defines the risks of malignancy. Statistics have shown that a new ovarian cancer occurs in every 10,000 women with ovarian endometriosis. Moreover, the probability of developing an ovarian cancer during the life of the woman with endometrioma increases from 1/100 to 2/100. An untreated woman has a 98% chance of not developing ovarian cancer instead of 99%.

#### Hormonal Treatments Available

The lesion profile is intended to assist in the selection of hormone therapy. It is actually recommended not to give progestogen if the lesions do not have a progesterone receptor (19). Different algorithms are possible to frame a laparoscopic surgery and ART (Figure 2). GN-RH agonists at a dose of 11.25 mg put the ovaries to rest and to suppress estrogen secretion for at least 3 months, up to 6 months. Moreover, the risk of osteoporosis is great and time is valuable. Laparoscopy with its staging informs us about the condition of the pelvis and the size of the lesions, so as to not let pass the moment of the ART (Figure 2). The 3-mg agonists are used for ovarian stimulation of ART. There is a molecule (Dienogest) which has a definite progestational effect on lesions. It is used while waiting for ART (11). Furthermore, the most useful progestin appears to be levonorgestrel. When contraception should be prescribed, it is recommended that it be combined with an estrogen. Finally, in case of dysmenorrhea, adenomyosis, a progestin-based IUD, is recommended.

## Conclusion

Endometriosis is a common disease that needs to be looked for and treated. Patients should be diagnosed early with pelvic pain and dysmenorrhea. The determination of blood and endometrial markers should allow a non-invasive and easily reproducible diagnosis.

Laparoscopy must be effective without becoming invasive and the time of ART must be considered early (Figure 2).

## Author Contributions

All authors listed have made a substantial, direct and intellectual contribution to the work, and approved it for publication.

### Conflict of Interest Statement

The authors declare that the research was conducted in the absence of any commercial or financial relationships that could be construed as a potential conflict of interest.

## References

[1] Acosta AA Buttram VC Besch PK Malinak LR Franklin RR Vanderheyden JD. A proposed classification of pelvic endometriosis. Obstet Gynecol. (1973) 42:19. 4720202

[2] American Fertility Society Revised American Fertility Society classification of endometriosis. Fertil Steril. (1985) 43:351 10.1016/S0015-0282(16)48430-X 3979573

[3] Bouquet de Joliniere J Effect of radiofrequency fields on endometriotic cell growth *in vitro*: a prospective study for laparoscopic treatment. J Minim Invasive Gynecol. (2007) 14:S14–5. 10.1016/j.jmig.2007.08.038

[4] Bouquet de Joliniere J Lesec G Real C Ayoubi JM Novel molecular strategy tests, issued by laparoscopy, that can predict the progression and severity of endometriotic disease. J Minim Invasive Gynecol. (2011) 18:S47–70. 10.1016/j.jmig.2011.08.174

[5] Fadhlaoui A Bouquet de Joliniere J Feki A. Endometriosis and infertility: how and when to treat? Front Surg. (2014) 1:24. 10.3389/fsurg.2014.00024 25593948 PMC4286960

[6] Bouquet de Joliniere J Ayoubi JM Gianaroli L Dubuisson JB Gogusev J Feki A. Endometriosis: a new cellular and molecular genetic approach for understanding the pathogenesis and evolutivity. Front Surg. (2014) 1:16. 10.3389/fsurg.2014.00016 25593940 PMC4286973

[7] Bouquet de Joliniere J Ayoubi JM Lesec G Validire P Goguin A Gianaroli L Identification of displaced endometrial glands and embryonic duct remnants in female fetal reproductive tract: possible pathogenic role in endometriotic and pelvic neoplastic processes. Front Physiol. (2012) 3:444 10.3389/fphys.2012.00444 23227010 PMC3512110

[8] Fadhlaoui A Gillon T Lebbi I Bouquet de Joliniere J Feki A. Endometriosis and vesico-sphincteral disorders. Front Surg. (2015) 2:23. 10.3389/fsurg.2015.00023 26157800 PMC4476201

[9] Carbonnel M N'guyen HT Abbou H Bouquet de la Joliniere J Ayoubi JM Robotic laparoscopy in benign gynecologic surgery: a retrospective study comparing vaginal, laparoscopic and robotic hysterectomy procedures. Reprod Syst Sex Disord. (2013) 2:1–5.

[10] Tran DK Belaisch J Is it the time to change the ASRM classification for endometriosic lesions? Proposal for a functional FOATIaRVS classification. Gynecol Surg. (2012) 9:369–73. 10.1007/s10397-012-0739-3

[11] Bouquet de Jolinière J Feki A Ayoubi JM The key points in gynecology: endometriosis, an atypical and topical disease. Régimédia. (2014) 1:17–24.

[12] Wicks MJ Larson CP. Histologic criteria for evaluating endometriosis. Northwest Med. (1949) 48:610–1. 18140833

[13] American Fertility Society Classification of endometriosis. Fertil Steril. (1979) 32:633 10.1016/S0015-0282(16)44409-2 510564

[14] Canis M Bouquet de Joliniere J Wattiez A Pouly JL Mage G Manhes H. Classification of endometriosis. Baillieres Clin Obstet Gynaecol. (1993) 7:759–74. 10.1016/S0950-3552(05)80462-6 8131314

[15] Tran DK Belaish J Classification of Endometriosis. Vth World Congress on Endometriosis. Yokohama: Parthenon Publishing Group (1991–1992) p. 259–67.

[16] Gogusev J Bouquet de Joliniere J Telvi L Doussau M du Manoir S Stojkoski A. Genetic abnormalities detected by comparative genomic hybridization in a human endometriosis-derived cell line. Mol Hum Reprod. (2000) 6:821–7. 10.1093/molehr/6.9.821 10956554

[17] Gogusev J Bouquet de Joliniere J Telvi L Doussau M du Manoir S Stojkoski A. Cellular and genetic constitution of human endometriosis tissues. J Soc Gynecol Investig. (2000) 7:79–87. 10.1177/107155760000700201 10785606

[18] Gogusev J Bouquet de Joliniere J Telvi L Doussau M du Manoir S Stojkoski A. Detection of DNA copy number changes in human endometriosis by comparative genomic hybridization. Hum Genet. (1999) 105:444–51. 10.1007/s004390051129 10598811

[19] Bouquet de Joliniere J Validire P Canis M Doussau M Levardon M Gogusev J. Human endometriosis-derived permanent cell line (FbEM-1): establishment and characterization. Hum Reprod Update. (1997) 3:117–23. 10.1093/humupd/3.2.117 9286736

[20] Simoens S Dunselman G Dirksen C Hummelshoj L Bokor A Brandes I. The burden of endometriosis: costs and quality of life of women with endometriosis and treated in referral centres. Hum Reprod. (2012) 27:1292–9. 10.1093/humrep/des073 22422778

[21] Huffmann JW External endometriosis. Am J Obstet Gynecol. (1951) 62:1243 10.1016/0002-9378(51)90050-6 14885314

[22] Kitsner RW Siegler AM Berhman SJ Suggested classification for endometriosis: relationship to infertility. Fertil Steril. (1977) 28:1008 10.1016/S0015-0282(16)42807-4 892039

[23] Buttram VC. An expanded classification of endometriosis. Fertil Steril. (1978) 30:240–42. 10.1016/S0015-0282(16)43467-9 680201

[24] Adamson GD Pasta DJ. Endometriosis fertility index: the new, validated endometriosis staging system. Fertil Steril. (2010) 94:1609–15. 10.1016/j.fertnstert.2009.09.035 19931076

[25] Palmisano GP Adamson GD Lamb EJ. Can staging systems for endometriosis based on anatomic location and lesion type predict pregnancy rates? Int J Fertil Menopausal Stud. (1993) 38:241–9. 8401684

[26] Vercellini P Fedele L Aimi G Pietropaolo G Consonni D Crosignani PG. Association between endometriosis stage, lesion type, patient characteristics and severity of pelvic pain symptoms: a multivariate analysis of over 1000 patients. Hum Reprod. (2007) 22:266–71. 10.1093/humrep/del339 16936305

[27] Adamson GD. Endometriosis classification: an update. Curr Opin Obstet Gynecol. (2011) 23:213–20. 10.1097/GCO.0b013e328348a3ba 21666464

[28] De Conto E Matte Ú Bilibio JP Genro VK Souza CA Leão DP Endometriosis-associated infertility: GDF-9, AMH, and AMHR2 genes polymorphisms. J Assist Reprod Genet. (2017) 1:P12–20. 10.1007/s10815-017-1026-z PMC5714819 28831646

[29] Bulun SE Yang S Fang Z Gurates B Tamura M Sebastian S. Estrogen production and metabolism in endometriosis. Ann N Y Acad Sci. (2002) 955:75–85; discussion 86-8:396–406. 10.1111/j.1749-6632.2002.tb02767.x 11949967

[30] Nephew KP Ray S Hlaing M Ahluwalia A Wu SD Long X. Expression of estrogen receptor coactivators in the rat uterus. Biol Reprod. (2000) 63:361–7. 10.1095/biolreprod63.2.361 10906038

[31] Taylor RN Vigne JL Zhang P Hoang P Lebovic DI Mueller MD. Effects of progestins and relaxin on glycodelin gene expression in human endometrial cells. Am J Obstet Gynecol. (2000) 182:841–7; discussion 847–9. 10.1016/S0002-9378(00)70333-4 10764460

[32] Zeitoun K Takayama K Sasano H Suzuki T Moghrabi N Andersson S. Deficient 17beta-hydroxysteroid dehydrogenase type 2 expression in endometriosis: failure to metabolize 17beta-estradiol. J Clin Endocrinol Metab. (1998) 83:4474–80. 10.1210/jcem.83.12.5301 9851796

[33] Béliard A Noël A Foidart JM. Reduction of apoptosis and proliferation in endometriosis. Fertil Steril. (2004) 82:80–5. 10.1016/j.fertnstert.2003.11.048 15236993

[34] Guidice LC Conti M. Growth factors and the development and function of the reproductive organs. Semin Reprod Med. (2009) 27:3–4. 10.1055/s-0028-1108004 19197799

[35] Cheng Y Li L Wang D Guo Q He Y Liang T. Characteristics of human endometrium-derived mesenchymal stem cells and their tropism to endometriosis. Stem Cells Int. (2017) 2017:4794827. 10.1155/2017/4794827 28761446 PMC5518492

[36] Latowska AM Lillington MD Shelling AN Cooke I Gibbons B Young BD FiSH analysis using cosmid probes to define chromosome 6q abnormalities in ovarian carcinoma cell lines. Cancer Genet Cytogenet. (1998) 77:99–105. 10.1016/0165-4608(94)90222-4 7954328

[37] Young VJ Brown JK Maybin J Saunders PT Duncan WC Horne AW. Transforming growth factor-β induced Warburg-like metabolic reprogramming may underpin the development of peritoneal endometriosis. J Clin Endocrinol Metab. (2014) 99:3450–9. 10.1210/jc.2014-1026 24796928 PMC4207934

[38] Tapper J Sarantaus L Vahteristo P Nevanlinna H Hemmer S Seppala M Genetic changes in inherited and sporadic ovatian carcinoma by CGH: extensive similarity except for a difference at chromosome 2q24-q32. Cancer Res. (1998) 58:2715–9. 9661879

[39] Kallioniemi A Kallionemi OP Sudar D Rutowitz D Gray JW Waldman F. Comparative genomic hybridization for molecular cytogenetic analysis of solid tumors. Science. (1992) 258:818–21. 10.1126/science.1359641 1359641

[40] Vinatier D Dufour P Oosterlynck D. Immunological aspects of endometriosis. Hum Reprod Update. (1996) 2:371–84. 10.1093/humupd/2.5.371 15717437

[41] Halme J Becker S Wing R. Accentuated cyclic activation of peritoneal macrophages in patients with endometriosis. Am J Obstet Gynecol. (1984) 148:85–90. 10.1016/S0002-9378(84)80037-X 6691385

[42] Jafarabadi M Salehnia M Sadafi R. Evaluation of two endometriosis models by transplantation of human endometrial tissue fragments and human endometrial mesenchymal cells. Int J Reprod Biomed. (2017) 15:23–32. 10.29252/ijrm.15.1.21 28280797 PMC5340136

[43] Iwabuchi T Yoshimoto C Shigetomi H Kobayashi H. Oxidative stress and antioxidant defense in endometriosis and its malignant transformation. Oxid Med Cell Longev. (2015) 2015:848595. 10.1155/2015/848595 26185594 PMC4491397

[44] Wu G Bersinger NA Mueller MD von Wolff M. Intrafollicular inflammatory cytokines but not steroid hormone concentrations are increased in naturally matured follicles of women with proven endometriosis. J Assist Reprod Genet. (2017) 34:357–64. 10.1007/s10815-016-0865-3 28074436 PMC5360689

[45] Da Broi MG Navarro PA. Oxidative stress and oocyte quality: ethiopathogenic mechanisms of minimal/mild endometriosis-related infertility. Cell Tissue Res. (2016) 364:1–7. 10.1007/s00441-015-2339-9 26685866

[46] Miller JE Ahn SH Monsanto SP Khalaj K Koti M Tayade C. Implications of immune dysfunction on endometriosis associated infertility. Oncotarget. (2017) 8:7138–47. 10.18632/oncotarget.12577 27740937 PMC5351695

[47] Mitchell GW Farber M Medical versus surgical management of endometriosis. In: Reed D Christian D Saunders W, editors. Controversies in Obstetrics and Gynecology, vol 2 Philadelphia: WB Saunders (1974). p. 629.

